# The potassium channel *Kcne3* is a VEGFA-inducible gene selectively expressed by vascular endothelial tip cells

**DOI:** 10.1007/s10456-019-09696-8

**Published:** 2019-11-21

**Authors:** Ron A. Deckelbaum, Ivan B. Lobov, Eunice Cheung, Gabor Halasz, Saathyaki Rajamani, Julia Lerner, Chunxiang Tong, Zhe Li, Patricia Boland, Melissa Dominguez, Virginia Hughes, George D. Yancopoulos, Andrew J. Murphy, Gavin Thurston, Jingtai Cao, Carmelo Romano, Nicholas W. Gale

**Affiliations:** 1 Department of Pre-Therapeutic Target Discovery, Regeneron Pharmaceuticals Inc, Tarrytown, NY 10591 USA; 2 Department of Ophthalmology, Regeneron Pharmaceuticals Inc, Tarrytown, NY 10591 USA; 3 Department of Oncology & Angiogenesis, Regeneron Pharmaceuticals Inc., Tarrytown, NY 10591 USA; 4 Department of Bioinformatics, Regeneron Pharmaceuticals Inc, Tarrytown, NY 10591 USA; 5 Department of Research, Regeneron Pharmaceuticals Inc, Tarrytown, NY 10591 USA; 6 Palmira Biopharma, Moscow, 143026 RF Russia

**Keywords:** Endothelial tip-cell, *Kcne3*, VEGFA, Retinal angiogenesis

## Abstract

Angiogenesis is largely driven by motile endothelial tip-cells capable of invading avascular tissue domains and enabling new vessel formation. Highly responsive to Vascular Endothelial Growth-Factor-A (VEGFA), endothelial tip-cells also suppress angiogenic sprouting in adjacent stalk cells, and thus have been a primary therapeutic focus in addressing neovascular pathologies. Surprisingly, however, there remains a paucity of specific endothelial tip-cell markers. Here, we employ transcriptional profiling and a *lacZ* reporter allele to identify *Kcne3* as an early and selective endothelial tip-cell marker in multiple angiogenic contexts. In development, *Kcne3* expression initiates during early phases of angiogenesis (E9) and remains specific to endothelial tip-cells, often adjacent to regions expressing VEGFA. Consistently, *Kcne3* activation is highly responsive to exogenous VEGFA but maintains tip-cell specificity throughout normal retinal angiogenesis. We also demonstrate endothelial tip-cell selectivity of *Kcne3* in several injury and tumor models. Together, our data show that *Kcne3* is a unique marker of sprouting angiogenic tip-cells and offers new opportunities for investigating and targeting this cell type.

**Electronic supplementary material:**

The online version of this article (10.1007/s10456-019-09696-8) contains supplementary material, which is available to authorized users.

## Introduction

New blood vessel formation, or neovascularization, is a necessary physiological process that is highly coordinated with tissue growth and homeostasis, but is frequently dysregulated in disease states ranging from cancer to ischemia [1–3]. Angiogenesis, the primary mode of neovascularization, ensues through selection and sprouting of migratory endothelial cells (ECs) that break away from their stable positions within pre-existing blood vessels to form lumenized tubules that further remodel into an elaborate network of arteries, veins and capillaries [4, 5]. Residing at the leading edge of angiogenic sprouts, specialized endothelial tip cells (ETCs) retain migratory and invasive properties that allow for sprouts to reach the avascular tissue environment. ETCs express abundant levels of vascular endothelial growth-factor-A receptor 2 (VegfR2), and are highly responsive to the pro-migratory effects of VEGFA produced by tissues under oxygen and nutrient depravation [6]. Trailing behind ETCs are phenotypically distinct endothelial stalk cells (ESCs). Mediating vessel lumenization, ESCs are highly proliferative but exhibit reduced VEGFA-responsiveness and strongly express the Delta-like 4 (Dll4) receptor Notch1 [7, 8]. In a negative feedback loop, VEGFA stimulation of Dll4 expression in ETCs results in Notch1 activation in subjacent ESCs, which in turn downregulate VegfR2 and promote pericyte recruitment and vessel maturation [9–11]. The balance between ETCs and ESCs is therefore of crucial importance to developmental and pathogenic angiogenesis, where even partial loss of VEGFA or Dll4 function results in blunted angiogenesis or exuberant endothelial sprouting, respectively [12–14]. Despite the fact ETCs have been a primary focus for targeted antiangiogenic therapies in tumors and vascular eye disease, there remains a surprising paucity of specific molecular markers for this cell type.

Distinguishing ETCs has historically relied on studies of retinal angiogenesis, where a vascular plexus develops from the central optic stalk that expands radially and superficially along the retinal inner neural surface (P0–P7 in mouse, [15]). This unique concentric planar organization allows for spatial localization of ETCs at the leading edge of the vascular plexus, where elaborate filopodia extensions distinguishes them morphologically. Current molecular identifiers of ETCs include the endothelial markers *Kdr* (VegfR2), *Flt4*, *Pdgfb*, *Dll4*, and *Unc5b*, which exhibit higher expression levels relative to ESCs but are not exclusive to ETCs [6, 8, 16–18]. For example, *Dll4* and *Unc5b* are expressed by both ETCs and by arterial endothelial cells, while *Flt4* is also expressed by venous endothelial cells—reflective of their functional roles in sprouting angiogenesis and vessel remodeling. More recently, interrogation of expression profiles from isolated retinal ECs led to the identification of ETC-enriched genes (e.g. *Cxcr4*, *Apln*, *Esm1*, and *Angpt2*), with the VEGFA-response gene *Esm1* validated by genetic fate mapping [19–23]. However, a number of these putative ETC markers are also active in other vascular compartments, and it is therefore unclear whether they confer ETC-specificity outside of retinal angiogenesis. Thus, the characterization of distinct ETC markers within multiple angiogenic contexts remains a pertinent objective.

*KCNE3*, formerly named Mink-related Peptide 2 (MiRP2), is a member of the KCNE family of ancillary β-subunits that assemble with α-subunits of voltage-gated potassium (K^+^) ion channels, including KCNQ1 and Kv3.4, and which functions in modulating their electrophysiological properties [24, 25]. In mice, *Kcne3* is reportedly expressed in the small intestine, colon, and trachea, where it localizes to the basolateral aspects of the mucosal epithelium [26]. Interestingly, *Kcne3* knockout mice are viable and fertile but exhibit perturbations in transepithelial transport of Cl^−^ and cardiac arrhythmias associated with adrenal-targeted autoimmune responses [26, 27]. However, expression of *Kcne3* in vascular endothelial cells has not been described. In the present study we interrogated transcriptomes from early postnatal mouse retinas subjected to transient VEGFA activation or blockade and reveal *Kcne3* as a robust endothelial-specific VEGFA-inducible gene. Utilizing a *Kcne3*–*lacZ* knock-in reporter mouse line, we show that, in vivo, *Kcne3* activation initiates by ETCs within multiple embryonic vascular plexuses (E9.5–E11.5), in close association with domains of VEGFA expression. *Kcne3* is not exclusively expressed in the vasculature: At later developmental stages and in the adult *Kcne3* is also expressed in the gut and tracheal epithelium.

Our studies provide first evidence that *Kcne3* may be exploited as a general ETC marker in multiple angiogenic contexts and opens new opportunities for targeting this specialized endothelial cell type for addressing neovascular disease states.

## Materials and methods

### Animal care and anesthesia

All animal studies were approved and performed in accordance with Regeneron’s Institutional Animal Care and Use Committee (IACUC) guidelines. For survival corneal surgeries, pups were anaesthetized using a mixture of oxygen and 2 volumes (%) of isoflurane employing the VETequip vaporizer. Local anesthesia to the eye was performed by applying a single drop of proparacaine.

### Intravitreal administration of VEGFA and VEGF-Trap

Intravitreal (ITV) microinjections (50–500 nl) were made between the equator and the corneal limbus using a Drummond nanoinjector equipped with a glass needle as described [9]. For this study, intravitreal injections were performed on 6-day old pups (P6), which received 4 μg of hFc (REGN379), 4 μg of VEGF-Trap (REGN3), or 1.5 μg of VEGFA_165_ (REGN110) per eye. The following day (P7), retinas were dissected and used for RNAseq or ISH/IB4 analyses.

### cRNAseq transcriptome analysis

Total RNA was purified using the MagMAX-96 for Microarrays Total RNA Isolation kit (Ambion) according to the manufacturer’s instructions, in which genomic DNA was removed using MagMAXTurboDNase buffer and Turbo DNase. mRNA was purified from total RNA using the Dynabeads mRNA purification kit (Invitrogen) according to the manufacturer’s instructions. For the P8 retina profiling, strand-specific RNA sequencing (RNA-Seq) libraries were prepared using the ScriptSeq mRNA-Seq Library Preparation kit (Epicentre). Twelve-cycle PCR was performed to amplify libraries. For the OIR study, cDNA was synthesized and amplified (12-cycle PCR) from ten nanogram total RNA using SMARTer^®^ Ultra^®^ Low RNA Kit (Clontech). Nextera XT library prep kit (Illumina) was used to generate the final sequencing library (12 PCR cycles performed to amplify libraries) using 1 ng of cDNA as the input. Sequencing was performed on an Illumina HiSeq 2000 instrument by a multiplexed, single-read run with 33 cycles. Raw sequence data (BCL files) were converted to Fastq format via Illumina Casava 1.8.2. Reads were decoded based on their barcodes, and read quality was evaluated using Fastqc (www.bioinformatics.babraham.ac.uk/projects/fastqc/). Reads were mapped to the mouse transcriptome (NCBI genome assembly GRCm38/mm10) using ArrayStudio’s OSA aligner, allowing for two mismatches. Exon mapped reads were summed at the gene level. Genes differentially expressed between hFc-, VEGFA-, or VEGF-Trap-treated samples were obtained using DeSeq2. A gene was considered regulated in a particular comparison if the nominal *P* value from DeSeq2 was less than 0.01 and if the mean expression increased or decreased by at least 50%.

### *Kcne3*–*lacZ* reporter mice

Employing Velocigene technology [28], a BAC construct was engineered to replace the entire coding sequence of the murine *Kcne3* gene with a β-galactosidase (lacZ)/floxed-neomycin cassette, inserted in-frame at the initiating ATG within exon 4. Homologous recombination within F1H4 ES cells (C57BL6/129SvJ hybrid), was followed by identification of correctly targeted ES cell clones using the Loss-Of-Allele Assay (LOA), [28]. Two independent ES clones were employed to generate chimeric mice that were subsequently bred to C57BL6 females to generate F_1_ mice, which were genotyped by LOA and verified histochemically for β-galactosidase activity. Heterozygous *Kcne3*–*lacZ* mice were maintained on a mixed genetic background (75% C57BL6/25% 129SvJ).

### LacZ reporter analysis

LacZ staining of whole embryos and retina was performed as previously described [29]. Briefly, dissected samples were fixed in 2% paraformaldehyde/0.2% glutaraldehyde for 1 h on ice, washes in PBS, and incubated with X-gal staining solution (5 mM K_3_Fe(CN)_6_, 5 mM K_4_Fe(CN)_6_, 2 mM MgCl_2_, 0.01% deoxycholate, 0.02% igepal, 1 mg/ml X-gal) for 12 h at 37 °C. Following post-fixation with 2% paraformaldehyde, specimens were washed in PBS and transferred into 70% glycerol/PBS prior for imaging. For histological preparations, samples were fixed as above, sunk into 15% and 30% sucrose, and OCT-embedded for cryosectioning. Following brief post-fixation, sections were stained with X-gal solution and counterstained with Neutral Red prior to mounting with DPX mounting media.

### Whole mount In Situ Hybridization and isolectin staining of retinas

A riboprobe encompassing 557 base pairs of the murine *Kcne3* coding sequence was amplified by reverse transcriptase (RT)-PCR (iScript cDNA Synthesis Kit, BioRad), employing the following primers: 5′-GAG ACT TCC AAC GGG ACT GA-3′, and 5′-CGC CAC AGC TTC CTC TTC-3′. This was subsequently cloned into the pGEMT-Easy vector (Promega), linearized with SacII, and mRNA transcription performed with SP6 polymerase and digoxigenin-labeled UTP using the MEGAscript kit (Ambion). Dissected retinas were fixed in 4% paraformaldehyde (PFA)/PBS for 16 h at 4 °C, gradually dehydrated into methanol and then rehydrated into PBS. Tissues were digested with proteinase K (10 μg/ml) for 20 min, and incubated in buffered detergent (1% NP-40, 1% SDS, 0.5% Na deoxycholate, 1 mM EDTA, 150 mM NaCl, 50 mM Tris, pH 8.0) for 30 min. Following post-fixation in 0.2% glutaraldehyde/4% PFA, retinas were placed in hybridization buffer (50% formamide, 5× SSC, 1% SDS, 50 μg/ml heparin, 200 μg/ml BSA, 100 μg/ml yeast RNA) for 60 min at 65 °C, and then overnight under similar conditions with the addition of *Kcne3* riboprobe. Unbound probe was removed by successive incubations with wash buffer (50% formamide, 1× SSC, 0.1% Tween 20) and a 1:1 mix of wash buffer-MABT (100 mM maleic acid pH 7.5, 150 mM NaCl, 0.1% Tween 20) at 65 °C. Following additional washes with MABT at room temperature, specimens were incubated with 15% normal goat serum/MABT for 2 h and then overnight with AP-conjugated anti-DIG (Roche, 1:5000). Following repeated washing with MABT, signal was detected using BM-Purple reagent (Roche). For subsequent vessel labeling, retinas were treated with biotinylated isolectin (Vector, #B1205; 1:200/PBST), washed several times in PBST and then with TBTI (50 mM Tris–HCl, pH 8.0, 150 mM NaCl, 0.1% Tween-20, 10 mM imidazole, 0.2% BSA). Fluorescent signal was detected using Cy3-tyramide (Perkin Elmer, #NEL744001KT) and 0.0015% hydrogen peroxide for 1 h.

### In Situ Hybridization by RNAscope on histological sections

Embryonic tissues were fixed in 4% PFA/PBS for 16 h and cryopreserved in OCT. Simultaneous detection of *Pecam1* and *Kcne3* mRNA was performed according to protocols developed by the manufacturer—Advanced Cell Diagnostics [30], using the following probes: Mm-Kcne3-C1, cat# 493831 and Mm-Pecam1-C2, cat#316721. Following all hybridization steps tyramide-488 amplification was employed to detect specific *Kcne3* signal, while *Pecam1* was detected using the RNAscope^®^ 2.0 HD Detection Kit (RED).

### Immunofluorescence on histological sections

Frozen OCT-embedded sections of mouse embryos were blocked for 1 h with 10% normal goat serum/PBST, incubated for 16 h at 4 °C with antibodies to β-galactosidase (Abcam, ab9361) and Pecam1 (BD Pharmingen, 553370). Immunoreactivity was determined using horseradish peroxidase conjugated donkey anti-chicken F(ab)_2_ (Jackson Immunolabs, 703-036-155) and donkey anti-rat (Jackson Immunolabs, 712-036-153), followed by sequential cy3-tyramide and 488-tyramide signal amplification.

### Oxygen-induced retinopathy (OIR) model

OIR was produced following the method developed by Smith et al. [31]. Briefly, litters of 6-day old (P6) mouse pups and their dams were placed in hyperoxia (75% oxygen) to induce capillary obliteration in the central retina. Eleven-day old pups (P11) were returned to room air and analyzed immediately or at P16. In a second study, P11 pups were subjected to normoxia until P15, at which time they received intraperitoneal injections of hFc or VEGF-Trap at 25 mg/kg. Retinas from the right eyes of these mice were assessed by fluorescein Griffonia Simplicifolia I (GS lectin I) (Vector Laboratories) staining of vessels and by NG2 (Millipore) with Alexa-Fluor 594 (Invitrogen) staining of neovascular tufts. Retinas from the left eyes were assessed by RNAseq transcriptomics 6- and 24-h following treatment (*N* = 4–5, per group).

### Corneal suture model

Employing adult *Kcne3*–*lacZ* mice, corneal sutures were placed in proximity to the central part of the cornea as previously described [32]. After 9 days, mice were euthanized and whole eyes were fixed in 4% PFA, washed in PBS, and stained in X-gal staining solution. Corneas were then sub-dissected, flat mounted, and imaged by light microscopy.

### LCC tumor model

Lewis Lung carcinoma cells (5 × 10^5^) were injected under the dorsal skin of syngeneic adult heterozygous or homozygous *Kcne3*–*lacZ* mice. Seven to ten days postimplantation a palpable tumor could be visualized under the skin at the injection site, at which time mice were euthanatized and tumors were processed for X-gal staining and imaging.

### ES tumor model

F1H4 ES cells were implanted sub-cutaneously into SCID mice as previously described [33]. Tumor growth was allowed to proceed until reaching 300–500 mm^3^, after which these were dissected, fixed in 4% paraformaldehyde, and processed for whole mount or histological X-gal staining. Representative sections were also stained for immunohistochemical detection of CD31 (Pecam1).

## Results

### *Kcne3* is induced by VEGFA in retinal vascular endothelial cells

**Fig. 1:**
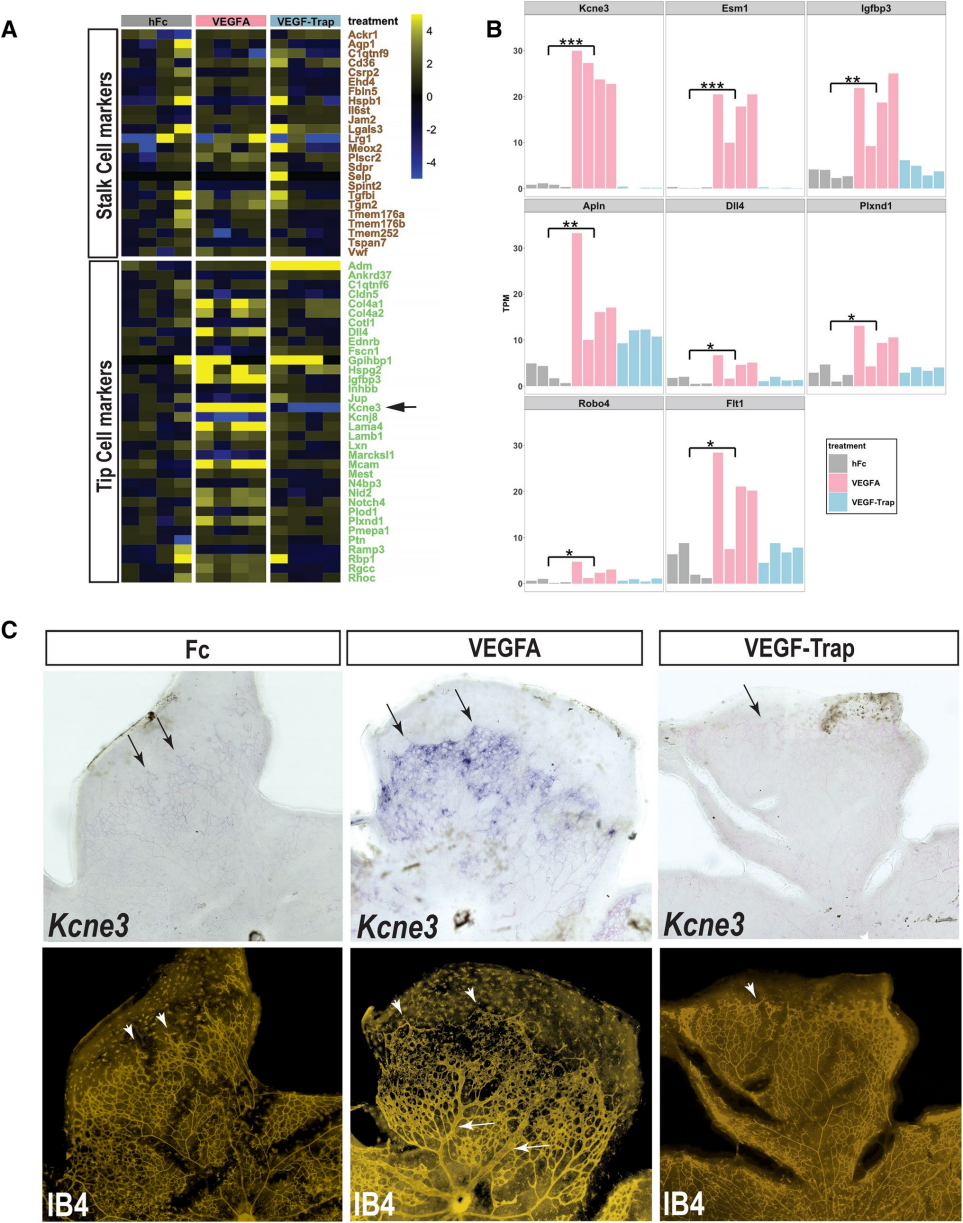
*Kcne3* is transcriptionally regulated by VEGFA in retinal endothelial cells. **a** Heat map displaying the expression profiles of previously defined endothelial stalk-cell or tip-cell genes, in postnatal day 8 (P8) mouse retinas 24-h following intravitreal injection of hFc, VEGFA, or VEGF-Trap. Scale shown is log_2_ transformed fold-change, relative to the median of hFc-treated controls. The analysis of *N* = 4 mice per treatment group is shown. **b** Comparative expression of *Kcne3* to cognate endothelial and ETC genes—*Esm1*, *Igfbp3*, *Apln*, *Dll4*, *Plxnd1*, *Robo4*, and *Flt1*, in retinas exposed to hFc, VEGFA, or VEGF-Trap. ***P_BH-corrected_ = 8 × 10^−40^ − 5 × 10^−38^, **P_BH-corrected_ = 4.6 × 10^−10^ − 1.7 × 10^−7^, *P_BH-corrected_ = 6.5 × 10^−4^ − 2.8 × 10^−3^. **c***Top panel*: whole mount ISH analysis of *Kcne3* mRNA expression in retinal preparations from P7 mice, 24-h following intravitreal injection of hFc, VEGFA, or VEGF-Trap (representative samples from *N* = 4 mice per treatment group). Weak *Kcne3* signal is observed in ETCs and ESCs within the leading angiogenic front of hFc-injected eyes (left panel, arrows), but is dramatically increased in VEGFA-injected eyes (middle panel, arrows). By comparison, no *Kcne3* signal is observed in VEGF-Trap-injected eyes (right panel)*. Lower panel*: sequential fluorescent isolectin (IB4-cy3) staining reveals significant vessel dilation in VEGFA-injected eyes (middle panel; arrows), and also shows that the majority of *Kcne3*+ cells localize to the peripheral aspects of the vascular plexus (middle panels, arrow and arrowheads)

We have previously shown that localized delivery of VEGFA to the developing postnatal retina rapidly and profoundly alters vessel morphogenesis, during which endothelial cells acquire a distinct transcriptional profile [34]. To better characterize the VEGFA-regulated endothelial transcriptome in vivo, we profiled by RNA-seq postnatal day 8 (P8) retinas 24 h following intravitreal administration of recombinant VEGFA_165_, human IgG1 (hFc), or VEGF-Trap (*N* = 4, per treatment group). A composite of VEGFR1 and VEGFR2 Ig domains fused to human Fc, VEGF-Trap effectively binds and neutralizes the activity of VEGFA, VEGFB, and placental-growth factor (PLGF) [35]. Compared to retinas from eyes injected with hFc protein, retinas exposed to VEGFA exhibited a robust signature consisting of 742 upregulated and 1280 downregulated genes (Fig. 1a, b and Supplemental Table 1), whereas VEGF-Trap injection resulted in 93 upregulated and 71 downregulated genes. To further examine VEGFA-induced effects on ETCs, we evaluated the expression of defined ETC and ESC signature markers derived from prior single-cell RNA-seq analyses of tumors [36]. Interestingly, many of the tumor-derived ETC markers were upregulated in retinas following transient VEGFA treatment, whereas ESC markers were largely unperturbed (Fig. 1a). Notably, VEGFA treatment resulted in the activation of previously reported ETC genes—*Esm1, Igfbp3, Dll4* [7, 19, 20]. Conspicuously, the retinal expression of one gene, *Kcne3*, closely mimicked that of the VEGFA-response gene *Esm1*, where a dramatic ~ 30-fold increase was observed following treatment with VEGFA (Fig. 1b). However, unlike most other putative ETC and generic EC genes that were upregulated by VEGFA (e.g. *Apln, Dll4, Plxnd1, Flt1*), the basal level of *Kcne3* was attenuated by VEGF-Trap, suggesting that *Kcne3* is a VEGFA-response gene in endothelial cells (Fig. 1a, b, supplemental Table 1).

We next sought to identify the cellular source of *Kcne3* mRNA by performing whole mount In Situ Hybridization (ISH) on retinas prepared 24 h after intravitreal delivery of VEGFA, VEGF-Trap, or hFc (*N* = 3–4). Chromogenic staining resulting from hybridization of the *Kcne3* riboprobe was examined in retinas co-labeled with isolectin-B4 (IB4), an agent used to specifically detect vascular endothelial cells [9]. Examination of hFc-injected eyes showed weak *Kcne3* signal co-localizing with IB4-positive ECs within the leading edge of the vascular plexus (Fig. 1c, left panels), but no signal was detected within the neural layers of the retina. Remarkably, VEGFA exposure dramatically increased the level of *Kcne3* expression within the vascular plexus, where strong staining was observed in both ETCs and ESCs but not in established arteries or veins (Fig. 1c, center panels). By comparison, *Kcne3* mRNA was not detected in retinas following delivery of VEGF-Trap (Fig. 1c, right panels). Our data therefore suggests that *Kcne3* is highly activated by VEGFA in retinal endothelial cells, prompting us to further investigate its specificity to ETCs.

### *Kcne3* is an ETC-specific gene during retinal angiogenesis

**Fig. 2:**
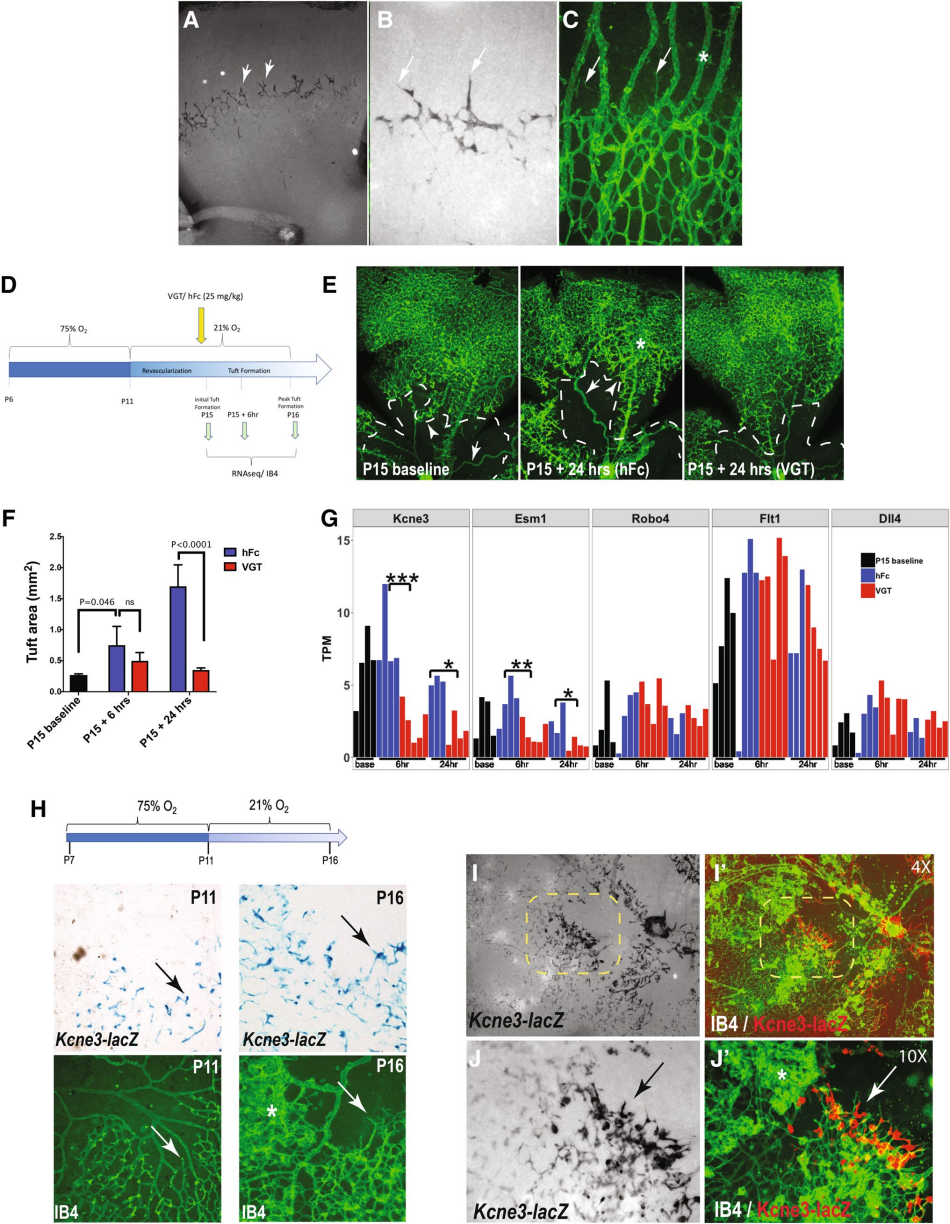
*Kcne3* is activated in ETCs during normal and pathogenic retinal angiogenesis. **a**–**c** β-galactosidase staining of P7 retina from heterozygous *Kcne3*–lacZ reporter mice at low and high-power showing specific expression in ETCs and weaker expression in ESCs along the angiogenic front (arrows **a**, **b**). **c** Comparative fluorescent isolectin (IB4) showing the overall vascular pattern compared to that in **b**; staining shows that *Kcne3*–*lacZ* is absent from hyaloid vessels (asterisk), but labels endothelial cells at the vascular front. **d** Schematic illustration of regimen for short-term OIR experiments. At the end of the hyperoxic phase (P11), single dose intravitreal injections of hFc or VEGF-Trap (VGT) was performed. Retinal samples were analyzed at baseline, 6-h, and 24-h from the start of neovascular tuft formation at P15 in normoxia. For each independent time point, analysis was performed on *N* = 3-5 mice per treatment group. **e** IB4-labeled retinal vessels at baseline and 24-h after return to normoxia, showing a reduction in the vaso-obliterative area (dotted line) and in neovascular tuft formation (asterisk) following VEGF-Trap delivery. **f** Quantitative image analysis of neovascular tuft area at baseline, 6-, and 24-h following return to normoxia. **g** Relative mRNA expression of selected endothelial markers, expressed as transcripts per million (TPM), derived from an RNAseq analysis of retinas subjected to OIR. Downregulation in expression levels of *Kcne3* and *Esm1* are observed under VEGF-Trap treatment conditions (compared to hFc), but other EC-specific genes are not significantly affected. **h** Analysis of *Kcne3*–lacZ reporter expression under standard OIR conditions (schematic). Comparative lacZ- and IB4-stained retina highlights the detection of *Kcne3* reporter activity in putative ETCs bordering the avascular zone that forms following a 4-day exposure to 75% O_2_, and which intensifies during 5-days upon return to normoxia (arrows). Note the absence of lacZ + cells in neovascular tufts (asterisk). **i**, **j**’ Low and high-power images of *Kcne3*–*lacZ* P16 retina, 5-days following the return to normoxia, detecting β-galactosidase and IB4+ vessels. *Kcne3*–*lacZ*^+^ ETCs inundate the vascular-avascular interface (arrows), but are largely absent from neovascular tufts (asterisk). ***P_value_ = 1.2 × 10^−5^, **P_value_ = 6.4 × 10^−5^, *P_value_ = 6.5 × 10^−4^

To better characterize endogenous *Kcne3* expression during normal phases of retinal development, we employed homologous recombination (VelociGene technology) to generate genetically modified mice expressing β-galactosidase (*lacZ*) in place of the *Kcne3* coding sequence (Suppl. Fig. 1). Mice heterozygous or homozygous for the *Kcne3*–lacZ allele are fully viable and fertile, and do not display overt vascular phenotypes (data not shown). Analysis of β-galactosidase activity in retinas of *Kcne3*–*lacZ* reporter mice at P7 revealed highly specific expression within the leading edge of the developing vascular plexus (Fig. 2a–c). Unlike Dll4, which is expressed by both ETCs and arterial endothelial cells [9, 16], *Kcne3*–*lacZ* remains restricted to ETCs and some ESCs of the angiogenic front but is absent from differentiated vessels (arteries, veins, hyaloid).

We next investigated *Kcne3* gene expression under conditions of pathologic retinal angiogenesis. The widely used oxygen-induced retinopathy (OIR) murine model recapitulates pathogenic aspects of neovascular retinopathies in humans. Here, vascular degeneration and ischemia within the central retina is induced by exposure of early postnatal mice (P6-P11) to high oxygen levels (75%), which upon return to normoxia manifest pathologic neovascular tuft invasion of the vitreal space—a phenomenon largely governed by dysregulated Vegfa expression [37]. To correlate between overt changes to vessel morphology and associated transcriptomic alterations under OIR conditions, at the beginning of neovascular tuft formation at P15, a single dose of hFc or VEGF-Trap was systemically delivered (Fig. 2d). Vessel analysis (IB4) and RNAseq were performed on retinal samples collected 6- and 24-h following hFc or VEGF-Trap administration (*N* = 3–5 per group per time point). While retina from hFc-injected mice exhibited prominent central vaso-obliteration and formation of neovascular tufts that increased in severity between baseline and 24-h (Fig. 2e, f), a smaller avascular area and an abatement of neovascular tuft formation was observed under VEGF-Trap conditions. Parallel interrogation of transcriptome profiles revealed that while the majority of endothelial-specific genes were not significantly influenced by the administered agents, the expression of *Kcne3* and *Esm1* were notably downregulated at 6- and 24-h following VEGF-Trap treatment (Fig. 2g, supplemental Table 2). Direct examination of *Kcne3*–*lacZ* reporter activity under conditions of OIR further revealed that *Kcne3* is active in endothelial cells surrounding the avascular zone by the end of the hyperoxic phase, but its levels increase considerably by 6-days following the return to normal oxygen levels (Fig. 2h). Interestingly, co-detection of *Kcne3*–*lacZ*/IB4 shows that Kcne3 activity is predominantly restricted to ETCs at the vascular-avascular interface but is largely absent from neovascular tufts (Fig. 2i, j). Together, these observations indicate that *Kcne3* is a specific ETC marker during normal and pathologic retinal angiogenesis, which can further expand into adjacent endothelial cell types under VEGFA excess.

### *Kcne3* is an ETC-specific gene during embryonic development

**Fig. 3:**
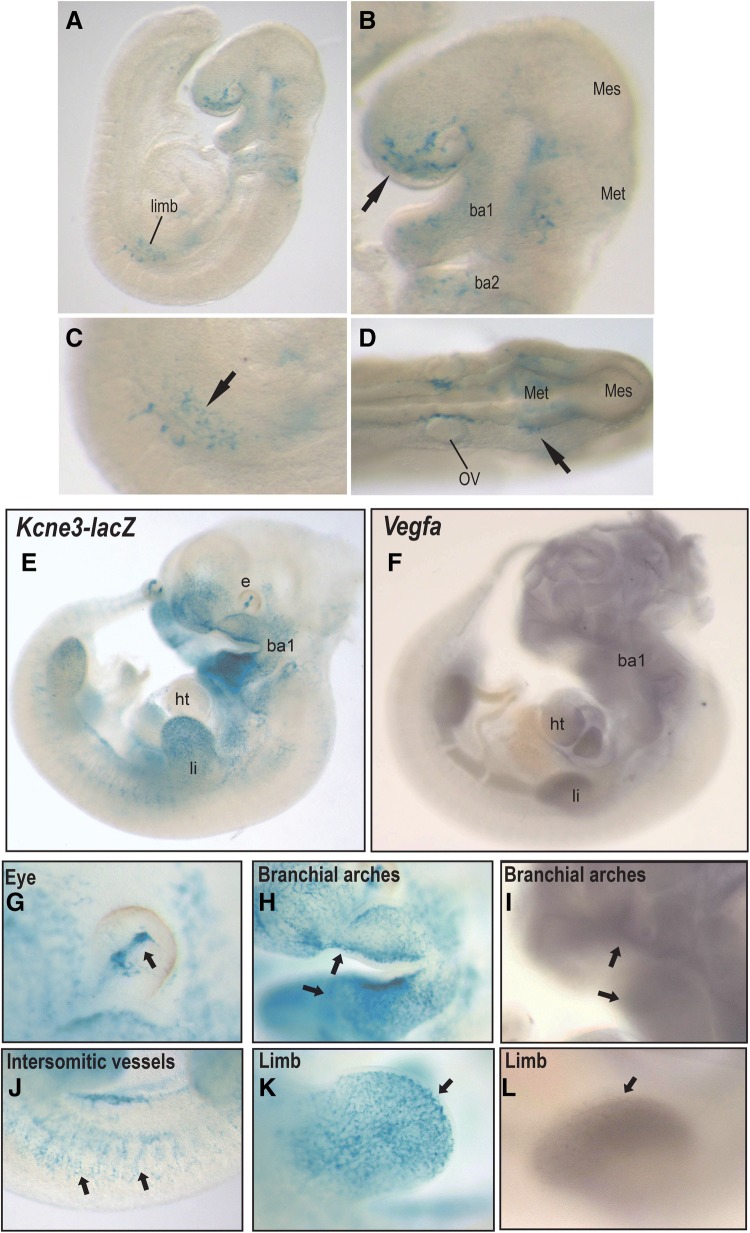
*Kcne3* is an early ETC marker during embryonic angiogenesis. **a**–**d** Analysis of β-galactosidase activity in homozygous *Kcne3*–*lacZ* embryos at E9.0 (21 somites), showing expression in nascent endothelial sprouts of the emerging forelimb bud (**a**, **c**), branchial arches and nasal prominence (B, arrow), and close proximity to mid- and hind-brain structures (**d** arrow). **e**–**l** Comparative of *Kcne3*–*lacZ* reporter activity and *Vegfa* mRNA (WMISH) in embryos at E10.5. *Kcne3*–*lacZ* reporter is specific to presumptive ETCs and endothelial sprouts forming the hyaloid vessels (**g**), microvasculature in branchial arches and facial prominence (**h**), intersomitic vasculature (**j**), and limb (**k**). Vegfa is detected in mesenchyme of all cephalic structures (**f**, **i**), heart (**f**), and limb (**l**). *e* eye, *li* limb, *ht* heart, *ba1* first branchial arch

**Fig. 4:**
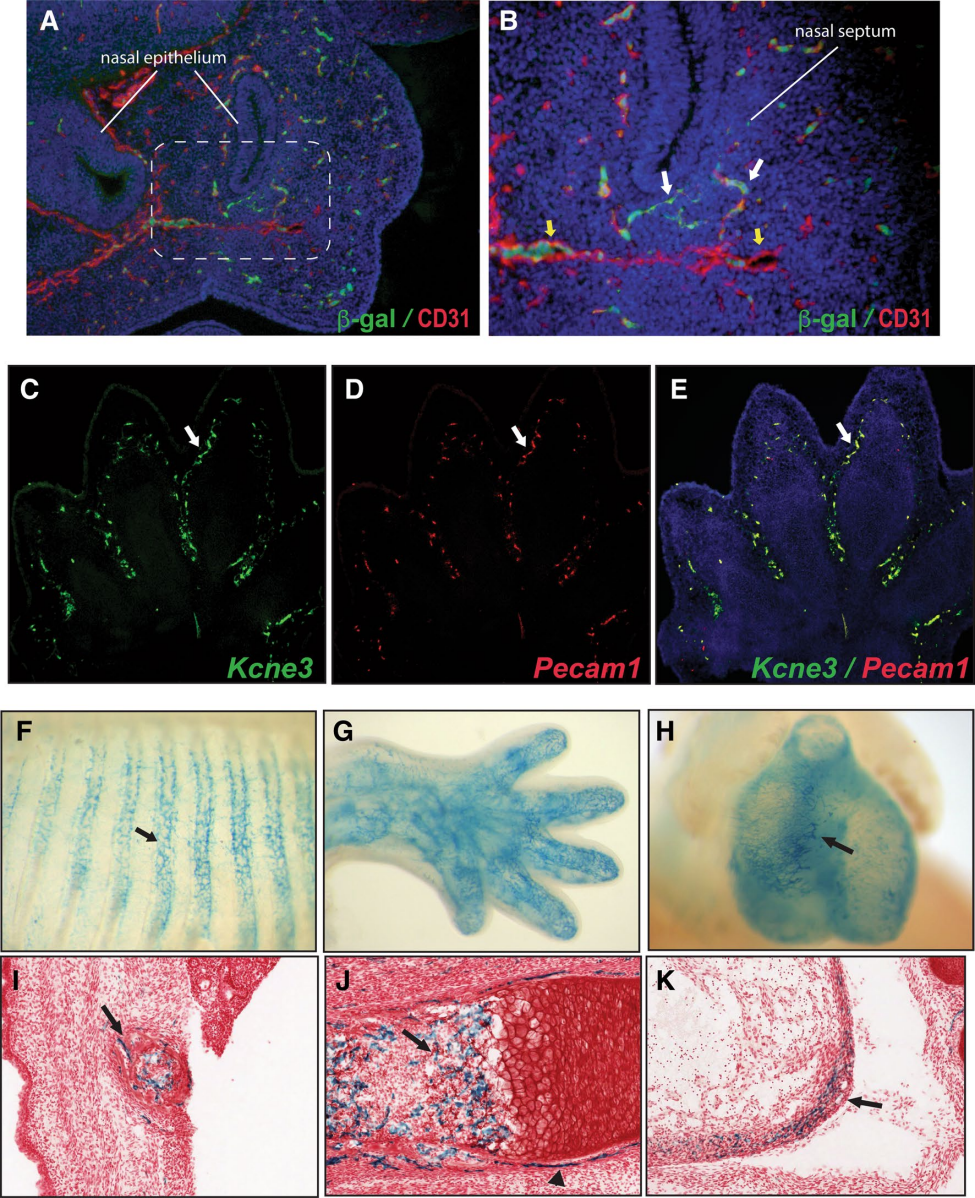
*Kcne3* localizes to ECs associated with the developing skeleton. **a**, **b** Immunodetection of β-galactosidase (green) and CD31 (red) within the nasal prominence of a *Kcne3*–*lacZ* embryo at E10.5 (“b” is a high-power view of dotted area in “a”). *Kcne3*–*lacZ*^+^ ECs are detected in microvascular sprouts within the condensing mesenchyme of the nasal septum (white arrows **b**), but are absent from lumenized vessels (yellow arrows **b**; Note: autofluorescent red blood cells are present in vessel lumen). **c**–**e** Double fluorescent In Situ Hybridization (ISH) employing RNAscope technology to simultaneously detect *Kcne3* (green) and *Pecam1* (red) mRNA in the distal limb of a wild-type mouse embryo at E13.5. *Kcne3* specifically localizes to all *Pecam1*^+^ ECs surrounding the cartilaginous digital elements. **f**–**k** β-galactosidase expression in *Kcne3*–*lacZ* mice at E15.5 showing robust staining in microvascular ECs surrounding skeletal anlagen including ribs (**f**, **i**), distal limb (**g**), and femur (**j**). At this stage, *Kcne3*–*LacZ*^+^ ECs are also in the coronary microvasculature of the heart (**h**, **k**)

To examine whether *Kcne3* may be a broad ETC marker in additional angiogenic contexts, we investigated *Kcne3*–*lacZ* expression dynamics during successive stages of mouse embryogenesis. In mouse, embryonic sprouting angiogenesis initiates shortly following the vasculogenic formation of primary aortic and venous structures (~ E7.5 to E8.5) [4, 38]. Comparatively, *Kcne3*–*lacZ* reporter activity first becomes evident at ~ E9.0 within sparse *lacZ*^+^ endothelial cells localizing to the facial and branchial prominences, intersomitic space, developing limb buds, and in juxtaposition to aspects of the neural tube (Fig. 3a–d). Interestingly, this nascent expression pattern significantly expands within 24-h (E10.5), where discernable *Kcne3*–LacZ-positive microvascular sprouts are present in the nasal prominence, branchial arches, limbs, hyaloid vessel of the eye, and portions of segmental vessels along the spinal column (Fig. 3e, g, h, j, k). As *Kcne3* is responsive to VEGFA in the retina, we sought to correlate its expression to that of embryonic *Vegfa* (E10.5). Interestingly, *Vegfa* expression as determined by WMISH, localizes to mesenchymal cells within or adjacent to *Kcne3 *+ domains within the facial prominences, branchial arches, and limb buds (Fig. 3f, i, l), as previously reported [39, 40]. Discerning the identity of *Kcne3*–*lacZ*^+^ cells, immunofluorescent analysis using anti-β-galactosidase and anti-CD31 (Pecam1)-specific antiserum shows that reporter activity is restricted to putative ETCs, but absent from the endothelium of lumenized vessels (Fig. 4a, b). Thus, *Kcne3* activation initiates within sprouting microvessels during early phases of embryonic angiogenesis and in close spatial and temporal association with *Vegfa*.

Interestingly, *Vegfa* originating from the primordial appendicular skeleton has been shown to critically regulate vascular morphogenesis in the limb [39]. Using RNAscope technology, double In Situ Hybridization analysis of *Kcne3* and *Pecam1* in the developing distal limb at E13.5, a stage at which chondrocytes begin to differentiate within the condensing digital mesenchyme, shows that *Kcne3* is expressed by all *Pecam1*^+^ endothelial sprouts surrounding the digital anlagen (Fig. 4c–e). In agreement, *Kcne3*–*lacZ* activity is remarkably pronounced within microvascular ECs closely associated with all endochondral skeletal elements by E15.5 (Fig. 4f, g, i, j), which abundantly populate both perichondrial and trabecular layers of the forming long bones (Fig. 4j). Outside of the skeleton at this stage, EC-specific *Kcne3*–*lacZ*^+^ activity is also observed within the coronary microvasculature (Fig. 4h, k), and capillaries of the esophagus, stomach, thymus, and dermis (Suppl. Fig. 2C, data not shown). Only rarely did we observe *Kcne3*–*lacZ*^+^ in lumenized arterioles or venules. Our data therefore indicates that during progressive stages of embryonic development *Kcne3* specifically localizes to ETCs within vascular beds undergoing active angiogenesis, but is never expressed by endothelial cells of established veins or arteries.

### *Kcne3* is specific to ETCs in during wound healing and tumor growth

**Fig. 5:**
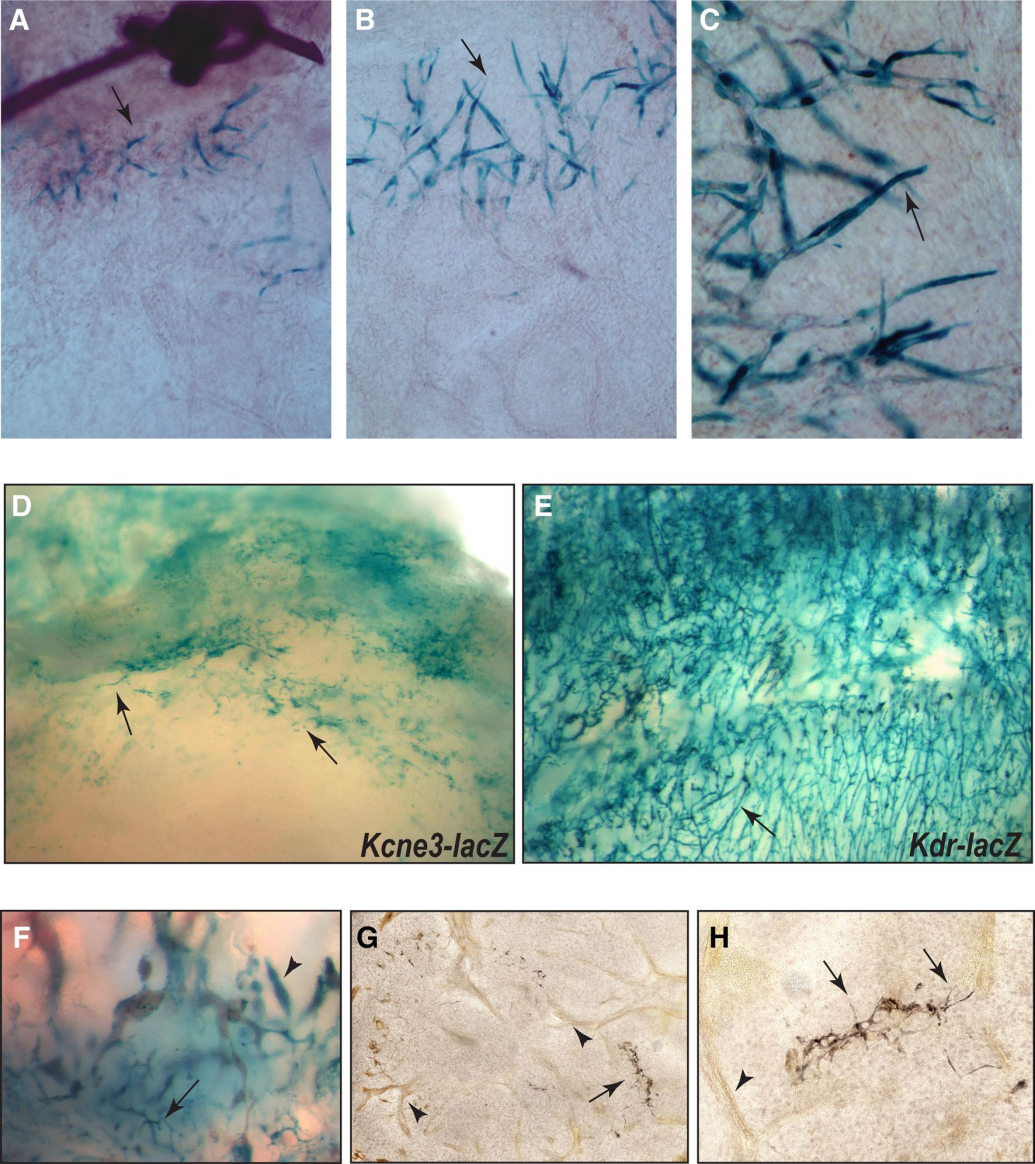
Kcne3 is expressed by endothelial tip cells during pathogenic angiogenesis. **a**–**c***Kcne3*–*lacZ* reporter expression in the corneal suture injury model. Seven days after suture insertion into the central cornea of *Kcne3*–*lacZ* reporter mice, β-galactosidase is detected in ETCs immediately adjacent to the suture implantation site (arrows), but not in any other vascular structures emanating from the limbal arcade. **d** LLC cells allografted subcutaneously into *Kcne3*–*lacZ* homozygous mice result in prominent host-derived lacZ expression within edges of the tumor microvasculature (**d**, arrows), but not in larger venules or arterioles. **e** LLC tumor allograft in *Kdr*-*lacZ* mouse showing broad expression throughout the capillary network (arrow). **f** Subcutaneous teratomas derived from *Kcne3*–*lacZ* ES cells showing specific β-galactosidase staining in microvascular (arrow) and epithelial structures (arrowhead). **g**, **h** Low- and high-power images of sectioned *Kcne3*–*lacZ* ES tumors showing the immuno-detection of CD31 in endothelial cells of lumenized blood vessels and capillaries (brown, arrowheads), while β-galactosidase is expressed solely by CD31^+^ ETCs (black, arrows)

As *Kcne3* is expressed by ETCs during normal development and is induced under oxygen deprivation, we also sought to investigate its activity within other models of pathogenic angiogenesis purportedly involving postnatal VEGFA function. The cornea serves as an excellent system for evaluating VEGFA-mediated pathogenic angiogenesis, where placement of sutures within the avascular corneal center promotes the invasion of blood and lymphatic vascular sprouts originating from preexisting vessels in the peripheral limbus [41, 42]. Applying this model to *Kcne3*–*lacZ* reporter mice, corneal tissues were examined by whole mount β-galactosidase staining 9-days following suture implantation. Strikingly, intense and specific lacZ + staining was observed at the leading edge of sprouting vessels within close proximity of the sutures, however, no staining was noted in the limbal vasculature (Fig. 5a–c, arrows). The morphology and positioning of stained endothelial cells suggests that these are comprised mostly of ETCs and some ESCs.

We next asked whether *Kcne3* is also upregulated during tumor angiogenesis. For this purpose, we evaluated β-gal expression in Lewis Lung Cell (LLC) tumor allografts introduced subcutaneously to homozygous *Kcne3*–*lacZ* mice. In this model, all β-gal expression is attributed to *Kcne3*–*lacZ* reporter activity originating from host-derived cells invading the tumor. Gross inspection shows robust and specific *Kcne3*–*lacZ* activity within capillary sprouts and putative ETCs that primarily localize to the tumor perimeter (Fig. 5d). In contrast, LLC tumors grafted onto *lacZ* reporters with global endothelial activity, as shown for *Kdr*-*lacZ*, exhibit broad capillary vessel staining that permeates the entire tumor (Fig. 5e). An additional tumor model we explored relies on the propensity of murine embryonic stem cells (ESCs) to form subcutaneous allograft teratomas, in which ESCs give rise to multiple cell lineages and tissue types [33]. Interestingly, ESC-derived endothelial cells have been shown to incorporate and contribute to the formation of the teratoma vasculature [33]. Allografted ESC clones harboring the *Kcne3*–*lacZ* allele into WT C57BL6 mice generated heterogeneous tumors that upon β-galactosidase staining revealed two discernable lacZ^+^ tissue types: epithelial and endothelial (Fig. 5f). Close examination of the tumor vasculature, however, shows that *Kcne3*–*lacZ* is specifically activated in endothelial cells localized to the microvasculature within peripheral aspects of the teratoma, which are revealed to be CD31^+^ ESC-derived ETCs (Fig. 5g, h). Notably, *Kcne3*–*lacZ* reporter activity was conspicuously absent from endothelial cells of lumenized vessels. Taken together, our data indicates that *Kcne3* is also a specific ETC marker during tumor angiogenesis.

### Expression of *Kcne3* in non-vascular tissues and organs

While initiating in embryonic microvascular endothelial cells, starting at E13.5 we also detected *Kcne3* mRNA in the gut epithelium by WMISH (Suppl. Fig. 2A, B). Indeed, examination of *Kcne3*–*lacZ* reporter mice shows that in addition to the intestine, expression prominently expands into the mucosal epithelial layers of the esophagus and stomach by E15.5 (Suppl. Fig. 2C–E, data not shown). By comparison to fetal stages, epithelial *Kcne3*–*lacZ* becomes more pronounced in adult organs. In agreement with prior reports, a cross-tissue examination at age of 12 weeks shows strong expression within the crypts of the small intestinal villi, and in the entire mucosal layer of the large intestine (Suppl. Fig. 3A, B; data not shown). In addition, however, our analysis reveals expression within organs and cell types not yet described. Surprisingly, robust epithelial *Kcne3*–*lacZ* staining is detected throughout the liver bile duct network, which further extends into the gallbladder proper (Suppl. Fig. 3B–F). Additional epithelial expression is observed within the trachea, salivary gland, lacrimal ducts, cutaneous sebaceous glands, pancreatic ducts (Suppl. Fig. 3G, H; data not shown). Although not characterized histologically, specific staining within mesenteric lymph nodes is suggestive of expression in immune cell types (Suppl. Fig. 3I). In addition, female mice exhibit distinct *Kcne3*–*lacZ* activity in ovaries and the oviduct (Suppl. Fig. 3J).

Consistent with expression in ETCs, which are largely absent in normal mature tissues, analysis of adult organs shows that by comparison to embryos, only a limited number of vascular beds exhibit *Kcne3*–*lacZ* expression. Here we observed expression in the arcuate arteries of the kidney, mesenteric vasculature, and peripheral arterioles within muscle (Suppl. Fig. 3K–N). Punctate lacZ staining is also present in growth plates of endochondral bone, but to a much lesser extent than observed in the fetal skeleton (Suppl. Fig. 3O, P). Thus, although *Kcne3* initiates as an ETC-specific gene during embryonic and retinal development, its postnatal extravascular expression likely reflects broader physiological functions.

## Discussion

A number of findings presented in this study indicate that, within the vasculature, *Kcne3* is a VEGFA-inducible gene selectively expressed by endothelial tip cells during normal and pathogenic angiogenesis. First, our transcriptome and spatial analyses of *Kcne3* localization within the retinal vascular plexus are consistent with published microarray data identifying *Kcne3* amongst genes upregulated in retinal ETCs [21]. Second, reporter allele activity confirms that *Kcne3* is specific to ETCs during developmental retinal angiogenesis, neovascular recovery following OIR, corneal injury, and in several tumor angiogenic models. While we postulate that ETC restriction of *Kcne3* is primarily regulated by astrocyte-sourced VEGFA during developmental retinal angiogenesis [6], a pattern also maintained along the vaso-obliterative perimeter in OIR, it is conspicuously excluded from pathologic neovascular tufts. Although the precise mechanism of neovascular tuft formation is not entirely clear, these consist of highly disorganized, poorly perfused, small-caliber vessels that lack identifiable tip cells [15]. It is therefore likely that *Kcne3* localizes to pre-tuft ETCs but is not induced within pathogenic non-tip endothelial cells, even in the presence of elevated VEGFA. Nonetheless, total VEGFA-blockade at this critical juncture both normalizes pathogenic angiogenesis and rapidly suppresses *Kcne3* transcription (most likely in bone fide ETCs).

Remarkably, intravitreal delivery of VEGFA during physiological retinal angiogenesis results in robust and immediate upregulation of *Kcne3* within the majority of ECs comprising the retinal vascular plexus (excluding those populating differentiated vessels), suggesting a broad potential by endothelial cells to respond to VEGFA. This is not entirely unexpected since ETC identity and phenotype, including filopodia formation and directionality, are dictated by fine VEGFA gradients emanating from concentric astrocytes [6]; overriding these by VEGFA excess, results in altered ETC distribution, filopodia misguidance, and overt alterations to vasoconstriction [6, 34]. Although our data strongly suggests that *Kcne3* is a VEGFA-inducible gene, additional molecular studies will be necessary to determine whether it is a direct or indirect target of VGEFA-VEGFR2 signaling in ECs. VEGFA-mediated receptor tyrosine kinase activation transduces multiple complex effector pathways, however, their associated transcription factors (e.g. SoxF, EST1) have only recently been recognized [52, 53]. Interestingly, a recent study identified *Kcne3* (and *Esm1*) amongst the top FoxO-regulated genes downstream of PI3-AKT, an effector pathway of VEGFR2, in lung and liver ECs [54]. Whether *Kcne3* is in fact regulated by FoxO1/3/4 in cis remains a compelling area of investigation that may provide answers on how this gene is transcriptionally regulated by VEGFA signal outputs.

In agreement, the temporal and spatial expression dynamics of *Kcne3* closely mimic that of *Esm1*, but not of other purported tip-cell genes (e.g. *Dll4*, *Angpt2*, *Cxcr4*). *Esm1* was initially identified as an endothelial-specific VEGFA target gene [43, 44], which in the retina is restricted to ETCs and in turn potentiates several aspects of VEGFA_165_ bioactivity [19, 20, 23]. Thus, both *Kcne3* and *Esm1* have been shown to be similarly suppressed in tumors responding to VEGFA-blockade [45]. However, in contrast to *Kcne3*, which appears to be a universal ETC marker in multiple angiogenic contexts (retina, embryo, tumor), *Esm1* loses ETC-specificity outside of the retina [20]. In fact, during embryonic angiogenesis, *Kcne3*–*lacZ* and mRNA closely apposes *Vegfa* expression domains—most notably in the developing limb and endochondral skeletal elements. This latter aspect is of particular interest as VEGFA production by the chondrogenic lineage is crucial for primordial limb angiogenesis [39], and the subsequent regulation of fetal and postnatal cartilage and bone formation [46–49]. The recent identification of a molecularly distinct EC subtype that supports osteoblastogenesis and matrix mineralization is consistent with *Kcne3 *+ ECs tightly associating with metaphyseal ossification centers, and thus may serve as a specific VEGFA-responsive and targetable marker for this unique cell population [50, 51]. Thus, in multiple contexts *Kcne3* shows characteristics of a bone fide ETC marker that is highly attuned to local VEGF-ligand concentrations.

Consistent with previously published data [26, 27], we find that homozygous *Kcne3*^*lacZ/lacZ*^ are fully viable and do not manifest overt phenotypes under non-challenged conditions. Excluding the possibility of subtle changes to angiogenesis, *Kcne3* mutants did not display obvious alterations to embryonic or retinal vessel morphology (not shown), suggesting that this gene is not required for vascular development. However, whether a functional role for *Kcne3* may be revealed under pathogenic neovascular settings remains to be investigated. The four classes of potassium channels comprised of 77 genes and 16 families, of which many associate with additional accessory β-subunits (e.g. Kcne3), ensures immense diversity and probable functional redundancy [55]. Recently, a functional role for the inwardly-rectifying potassium channel Kcnj2/Kir2.1 has been demonstrated in retinal vascular remodeling [56]. It is possible that a clearer biological role for *Kcne3* in endothelial cells would be elucidated with the combinatorial ablation of its primary channel components, Kcnq1 or Kcnc4. Alternatively, *Kcne3* may exert gain-of-function effects under conditions of ectopic overexpression, as mediated by VEGFA excess. In agreement, ectopic expression of Kcne3 in the heart was shown to alter the electrophysiological properties of ventricular contraction [57].

Beyond endothelial activation, our study revealed that *Kcne3* is subsequently expressed by multiple epithelial cell types. While *Kcne3*–*lacZ* reporter activity in the alimentary system (esophagus, stomach, intestine) is consistent with prior reports [26], its expression within hepatic bile duct network and gallbladder is suggestive of broader roles in maintaining electrophysiological properties across mucosal membranes. Hence, while we propose that the *Kcne3* allele can be effectively engineered for fate mapping the ETC lineage, as demonstrated for *Esm1* [23, 58], careful strategies should be considered when pursuing ETC-specific loss-of-function studies.

## Electronic supplementary material

Below is the link to the electronic supplementary material.

Electronic supplementary material 1 (AI 1857 kb)

Electronic supplementary material 2 (AI 34,641 kb)

Electronic supplementary material 3 (AI 33,977 kb)

Electronic supplementary material 2 (XLS 375 kb)

Electronic supplementary material 3 (XLS 284 kb)
